# Research on the Modulation Transfer Function Detection Method of a Bayer Filter Color Camera

**DOI:** 10.3390/s23094446

**Published:** 2023-05-01

**Authors:** Yuan-Peng Fan, Lei Wei, Lin Li, Lin Yang, Zi-Qiang Hu, Yuan-Hao Zheng, Yu-Hao Wang

**Affiliations:** 1 Institute of Frontier and Interdisciplinary Science, Shandong University, Qingdao 266237, China; fanyuanpeng@mail.sdu.edu.cn (Y.-P.F.); yanglincas@sdu.edu.cn (L.Y.); huziqiang20@mail.sdu.edu.cn (Z.-Q.H.); 202221101@mail.sdu.edu.cn (Y.-H.Z.); 202117041@mail.sdu.edu.cn (Y.-H.W.); 2 Space Optoelectronic Measurement and Perception Lab., Beijing Institute of Control Engineering, Beijing 100190, China

**Keywords:** modulation transfer function (*MTF*), Bayer filter, color camera, slanted-edge method

## Abstract

Bayer filter color cameras are more and more widely used in the field of aerospace remote sensing, but the Bayer filter causes great degradation in image quality; therefore, obtaining a means of achieving the high-precision measurement of the modulation transfer function (*MTF*) of Bayer filter color cameras is an urgent problem. In order to solve this problem, this paper develops a slanted-edge method via three steps: the detection of the slanted edge, the acquisition and processing of the edge spread function (ESF), and the acquisition and processing of the line spread function (LSF). A combination of the Canny operator and Hough transform is proposed for the detection of the slanted edge, which improves the fitting accuracy and anti-interference ability of the algorithm. Further, the Canny operator is improved by constructing an adaptive filter function and introducing the Otsu method, which can more effectively smooth the image and remove its false edges. A method of processing ESF data by combining cubic spline interpolation and Savitzky–Golay (SG) filtering is proposed, which reduces the effects of noise and the non-uniform sampling of ESF on *MTF*. A method of LSF processing using Gaussian function fitting is proposed to further reduce the effect of noise on *MTF*. The improved algorithm is verified by the *MTF* measurement test applied to a specific type of Bayer filter color space camera. The simulation and test results show that the improved slanted-edge method discussed in this paper has greater precision and a better anti-interference ability, and it can effectively solve the difficult problem associated with *MTF* detection in Bayer filter color space cameras.

## 1. Introduction

With the rapid development of remote sensing technology, the requirements made of color images are increasing. At present, there are mainly two ways to obtain color images. The first way is to use three charge-coupled devices (CCDs) or complementary metal-oxide semiconductors (CMOSs) to receive the three primary colors of incident light separately and then synthesize a color image. However, this approach is bulky, expensive, and difficult to install and adjust, so it is rarely used in space cameras. The second way is to use a camera featuring a Bayer filter, which can acquire color images using a single CCD or CMOS. Bayer filter color cameras acquire color images by adding a color filter array (CFA) arranged in a certain pattern on the detector surface to achieve color separation, according to which each pixel can only be sensitized to one primary color [1]. The other two primary colors are obtained by an interpolation algorithm, and the color image is then recovered. Bayer filter color cameras have the advantages of small size and low cost, and they are increasingly used in aerospace cameras. However, Bayer filter color cameras feature a significant degradation in image quality due to the reduced sampling rate, which requires a more accurate image quality evaluation method. Therefore, for Bayer filter color space cameras, the detection and evaluation of image quality with high accuracy after degradation is an urgent issue.

Both interpolation algorithms and color filter arrays can have an impact on imaging quality. There are various ways to evaluate imaging quality, and Bae TW proposed an evaluation system that assesses the imaging performance of color filter arrays [2]. In this paper, the modulation transfer function (*MTF*) is used as an evaluation indicator in order to achieve the fast detection of imaging quality to meet the demands of real-time focusing. *MTF* is an accurate and objective evaluation index of imaging quality, and it is one of the most important indicators of image quality [3,4,5]. The methods most commonly used to measure *MTF* are the slit method [6,7], the contrast method [8], and the slanted-edge method [9,10,11]. The measurement principle of the slit method is to obtain the *MTF* by Fourier transform of the line spread function (LSF) of the slit image. The calculation results derived at high frequencies via the slit method are more accurate, but its processing accuracy is left wanting. The principle of the contrast method is to form a stripe target of a specific frequency and then obtain the *MTF* at this frequency by calculating the contrast of the image. The calculation results of the contrast method are relatively accurate, but obtaining a continuous *MTF* curve requires multiple sets of expensive stripe plates, and the cost is very high. The slanted-edge method can calculate the response of the camera to different spatial frequencies using only a knife-edge target. The slanted-edge method can derive the *MTF* curve at a continuous spatial frequency, and the target is simple to make, so this process is the most widely used in the measurement of *MTF*. The International Organization for Standardization published the ISO 12233 protocol for electronic still image camera resolution testing in 2000, which defines the calculation process of the slanted-edge method in detail [12].

However, when the ISO 12233 method is used to measure the *MTF* of a Bayer filter color camera, the *MTF* curve is often aliased before the Nyquist frequency, which is caused by insufficient precision. There are three main reasons for this phenomenon: First, when performing the detection of the slanted edge, the algorithm is susceptible to noise due to its insufficient anti-interference capability. Second, since the angle of the slanted edge cannot be precisely controlled, the sampling of the edge spread function (ESF) is non-uniform, which will reduce the calculation accuracy of the *MTF*. Third, the acquisition of the LSF requires a differential operation, which further increases the influence of noise on the measurement results. Therefore, in order to solve these problems, this paper first analyzes the causes of image quality degradation in Bayer filter color cameras. Then, the slanted-edge method is developed in three main ways: the detection of the slanted edge, the acquisition and processing of the ESF, and the acquisition and processing of the LSF. The Canny operator [13,14] is improved using the method of constructing an adaptive filtering function and introducing the Otsu method [15] to enable the automatic optimal selection of scale parameters and thresholds. A combination of the improved Canny operator and the Hough transform is proposed for the detection of the slanted edge, which improves the anti-interference ability of the algorithm [16,17,18]. A combination of cubic spline interpolation and Savitzky–Golay (SG) filtering is proposed for ESF processing, which can be used to obtain uniform and smooth ESF data. A method of LSF processing using Gaussian function fitting is proposed to reduce the influence of noise on the *MTF* results. Finally, the improved slanted-edge method is used to complete the high-precision measurement of the *MTF* of a certain type of Bayer filter color space camera. The simulation and test results show that the improved slanted-edge method in this paper greatly improves the accuracy of the algorithm, and it can effectively solve the problem of the difficulty encountered in measuring the *MTF* of Bayer filter color cameras. In addition, some of the methods in this paper used for improving the slanted-edge method can also be extended to studies in other areas, such as turbulent and anisotropic media [19,20,21].

## 2. Causes of the Image Quality Degradation of Bayer Filter Color Cameras

The optical imaging system can be regarded as a linear space–time-invariant system, and the transfer function of each link in the imaging process reflects the attenuation of image details. The relationship between the overall *MTF* of the optical camera and the transfer function of each link can be expressed as

(1) $$MTF=MTF_{opt}\times MTF_{dec}\times MTF_{elec},$$

where *MTF_opt_*, *MTF_dec_*, and *MTF_elec_* represent the transfer functions of the optical lens, the detector, and the signal processing circuit system, respectively. In general, the drop in *MTF* caused by signal processing circuitry can be ignored. The *MTF* of an optical lens is related to the design and processing accuracy of the optical system, as well as the accuracy of assembly and adjustment. The optical system is kept as close to the diffraction limit as possible during design. Therefore, the overall *MTF* of an optical camera mainly depends on the choice of the detector [22].

**Figure 1:**
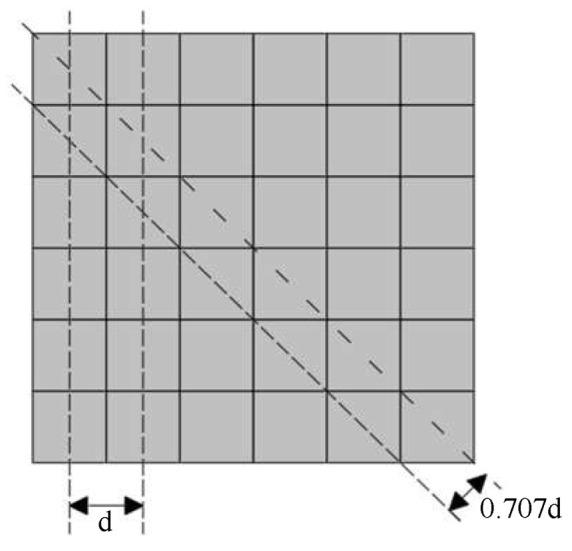
Schematic diagram and working principle diagram of the Bayer filter color detector.

The resolution of pixel-based detectors is limited, and the Nyquist sampling theorem stipulates that when the sampling frequency is greater than double the highest frequency, the sampling signal is able to completely retain the information of the original signal. The pixel distribution of the monochromatic detector is shown in Figure 1, and the total sampling frequency is composed of the sampling frequencies in the horizontal, vertical, and diagonal directions [23]. In Figure 1, *d* represents the interval between two pixels. It can be seen from Figure 1 that, compared with the horizontal and vertical directions, the diagonal direction is more able to display image details.

**Figure 2:**
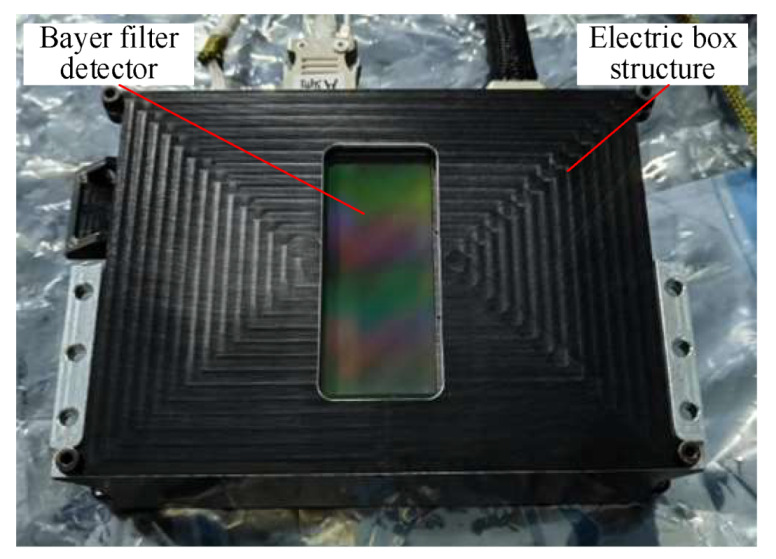
The focal plane component of the space camera used for *MTF* measurement in this paper.

**Figure 3:**
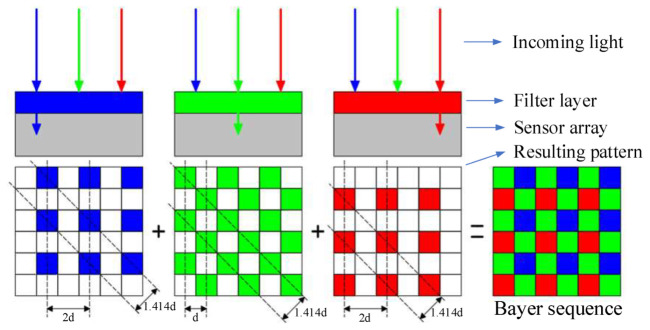
Schematic diagram and working principle diagram of Bayer filter color detector.

However, the resolution of the Bayer filter detector is significantly reduced compared to that of the monochromatic detector. The focal plane component of the space camera used for *MTF* measurement in this paper is shown in Figure 2, and the detector uses a Bayer-filtered detector. The working principle of the Bayer filter detector is shown in Figure 3. The front of the sensor is covered with an orderly distribution of CFA; the light passes through the filter array to reach the sensor, and every single pixel receives one color component of the three primary colors (red, green, and blue), while the gray value of the individual pixel represents the light intensity. Each pixel of the image obtained by the Bayer array has only one color gray value, and the process of using the surrounding pixels to estimate the two colors missing from the pixel in question is called color synthesis, also known as the demosaicing process. The most commonly used interpolation algorithms for color synthesis are the bilinear interpolation algorithm [24], the color smoothing interpolation algorithm [25], and the adaptive interpolation algorithm [26]. The bilinear interpolation algorithm is the most basic color synthesis algorithm and provides a good reference for other algorithms. In this paper, we will use the bilinear interpolation algorithm to synthesize color images and then measure the *MTF* of these images.

As can be seen in Figure 3, the sampling rate of the green channel in the horizontal and vertical directions shows the same frequency as the monochrome detector, but the sampling interval in the diagonal direction is doubled, thus reducing the sampling frequency. The blue and red channels are sampled at a lower rate than the green because the sampling intervals in the horizontal and vertical directions are also doubled. Therefore, the resolution of the synthesized color image is dramatically reduced due to the sharp reduction in the sampling rate of the original signal caused by the CFA, and the *MTF* of the synthesized color image is particularly susceptible to aliasing. Therefore, when measuring the *MTF* of an optical camera with a Bayer filter detector, the accuracy of the measurement algorithm must be higher, and the traditional slanted-edge method needs to be improved and optimized.

## 3. Improvement of the Slanted-Edge Method

**Figure 4:**
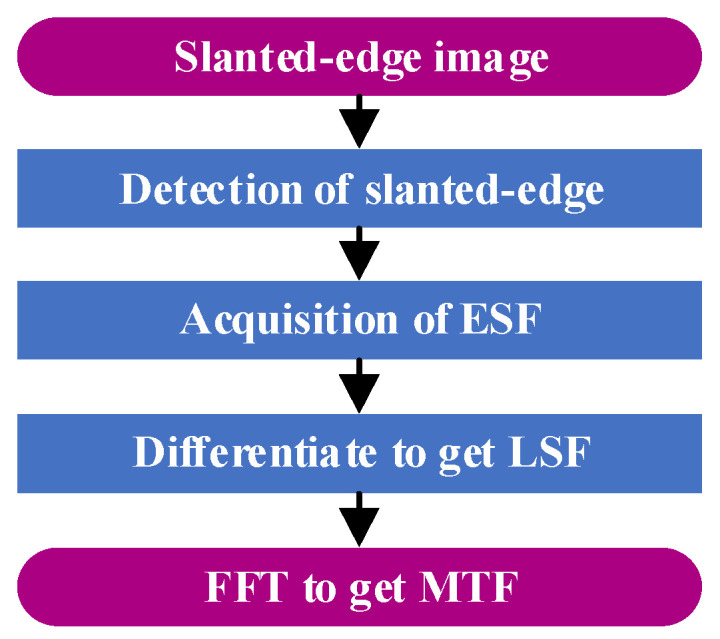
Basic process of edge method.

The basic process of the slanted-edge method is shown in Figure 4. The camera being assessed takes pictures of a knife-edge target to derive an image and then uses the slanted-edge detection algorithm to detect the slanted edge of the image. It uses the slanted-edge detection result to derive the edge spread function (ESF) and then uses the differential relationship between the ESF and line spread function (LSF) to derive the LSF. Finally, it performs fast Fourier transform (FFT) on the LSF to derive the *MTF*.

Only the basic steps of the slanted-edge method are introduced in the ISO 12233 publication. The analysis in the previous section shows that Bayer filter color cameras have higher requirements in terms of the accuracy of the algorithm when performing *MTF* measurements. Therefore, this paper optimizes and improves the three key steps of the detection of the slanted edge, the acquisition and processing of the ESF, and the acquisition and processing of the LSF.

### 3.1. Detection of Slanted-Edge

The detection of the slanted edge is an important part of the slanted edge method. The user must be able to accurately detect the position of the slanted edge of the image to ensure the accuracy of the subsequently acquired ESF and LSF and the calculation of *MTF*. The ISO12223 method uses the centroid method to calculate the position of the centroid of each row of pixels and then uses the least squares method to fit a straight line. The equation for the centroid method can be expressed as

(2) $$Centroid\left(j\right)=\frac{\sum_{i=1}^{i-1}i\times [g(i+1,j)-g(i,j)]}{\sum_{i=1}^{i-1}\left[g(i+1,j)-g(i,j)\right]}.$$

**Figure 5:**
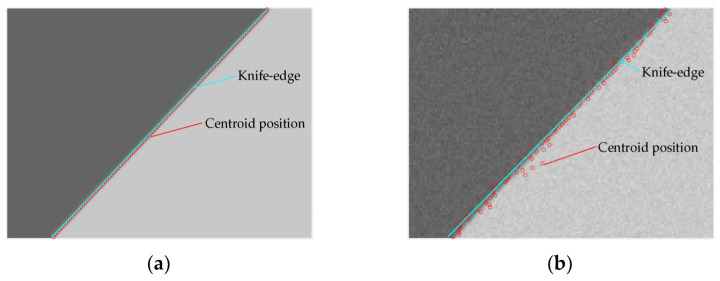
Simulation results of the ISO 12233 method used for the detection of slanted edges. (**a**) Results of the detection of a noiseless image. (**b**) Results of the detection of an image with noise.

In this equation, *i* and *j* represent the row and column of the image, respectively. *Centroid*(*j*) represents the position of the centroid of a certain row of pixels, and *g* represents the gray value of a certain pixel. When the image is not affected by noise, the ISO 12233 method yields a smaller error in the detection of the slanted edge. However, when there is noise in the image, the error of detection is relatively large due to the reduction in the calculation accuracy of the position of the centroid. The image simulated via the ISO 12233 method for the detection of the slanted edge is shown in Figure 5. The size of the image is 100 × 140 pixels. When generating a tilted edge, it is necessary to rotate the image, but it is difficult to accurately control and calculate the angle of the rotated edge. Therefore, we choose a slope of 1 to generate the simulation image. The digital number (DN) value of the bright edge is 200, and the DN value of the dark edge is 50. The degraded image is obtained using a Gaussian kernel function with a variance of 0.5 as a low-pass filter. Noise is added in Figure 5b, and the signal-to-noise ratio (SNR) of the image is 20 dB. When using the ISO 12233 method to detect the slanted edge of the image before and after adding noise, the fitting results of the slope are 1.0009 and 1.0232, respectively. It can be seen from the simulation results that the ISO 12233 method yields a small error when detecting the slanted edge of a noise-free image, and the result is poor when detecting the slanted edge of an image with noise.

The Hough transform is less sensitive to noise, and it is one of the main methods used for the detection of straight lines. The basic principle of the Hough transform is to transfer the detection of straight lines in image space to the detection of points in parameter space. The dual transformation of image space and parameter space can be expressed as

(3) ρ=xcosα+ysinα,

where *ρ* represents the vertical distance of the line to the origin, and *α* represents the angle between the *x*-axis and the vertical line of the line.

**Figure 6:**
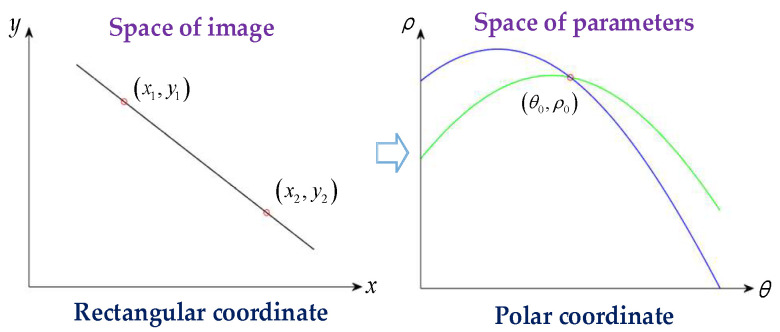
Principle of Hough transform for line detection.

As shown in Figure 6, this paper uses the Hough transform to transfer the detection of a straight line in the rectangular coordinate system to the detection of a point in the polar coordinate system. The points on a straight line in the rectangular coordinate system correspond to curves intersecting at a point in the polar coordinate system, and the line corresponding to the slanted edge is the point with the highest number of intersections in the polar coordinate space. The Hough transform needs to traverse all pixels, and *α* needs to be calculated using Equation (3) within the range of [−90°, 90°]. The smaller the step size of *α*, the higher the accuracy of line detection. Therefore, the Hough transform involves a high burden of calculation and takes a long time. In order to solve this problem, in this paper, we use the Canny operator to preprocess the image, preliminarily determine the location of the slanted edge, and improve the speed and accuracy of the Hough transform for line detection.

The preprocessing of the image by the Canny operator proceeds via four main steps: the smoothing of the image, the calculation of the gradient magnitude and direction, the suppression of gradient magnitude, and the removal of false edges. The Canny operator uses Gaussian filtering to smooth the image, and the variance is the scale parameter of the Gaussian filtering, which determines the smoothness of the Gaussian filtering window. The selection of the scale parameter is very important. The smaller the scale parameter, the higher the positioning accuracy, but the worse the noise processing effect. With a larger scale parameter, although the noise in the image can be better processed, the information of the slanted edge will become a high-frequency signal and will be easy to lose. When the Canny operator uses the non-maximum value to suppress the gradient magnitude, the selection of the threshold parameter is very important. If the threshold parameter is too high, the information on the slanted edge will be lost, and if it is too low, more false edges will appear. Therefore, in order to improve the accuracy of the Canny operator for image preprocessing, this paper proposes to use the adaptive function and Otsu method to achieve the optimal selection of scale and threshold parameters.

When the pixel point is a noise point, the scale parameter should take a larger value for smoothing; when the pixel point is a smooth area point, the scale parameter should take a smaller value to make the gray value change less; when the pixel point is a slanted-edge, it should lie between the above two cases. Therefore, the adaptive function is constructed in this paper as follows:

(4) $$\sigma_{best}=\left|g\left(i,j\right)-\frac{1}{m\times n}\sum_{x=-(m+1)/2}^{(m+1)/2}\sum_{y=-(n+1)/2}^{(n+1)/2}g\left(x,y\right)\right|,$$

where *σ_best_* represents the optimal scale parameter corresponding to the current pixel point, *g*(*i, j*) is the gray value of a current pixel point, and *m* and *n* represent the sizes of the filtering window. The basic principle of the Otsu method is to divide the image into two categories according to the gray features of the image and to determine the optimal threshold for suppressing the gradient amplitude in the Canny operator by finding the maximum interclass variance. Suppose the grayscale range of the image is {1, 2, …. *v*}, and suppose the optimal threshold is *H*. We divide the pixels into two classes, *C*_1_ = {1, 2, …. *H*} and *C*_2_ = {*H* + 1, *H* + 2, *…. v*}, and the interclass variance of *C*_1_ and *C*_2_ can be expressed as

(5) $$IV=p_{1}{\left(\mu_{1}-\mu_{0}\right)}^{2}+p_{2}{\left(\mu_{2}-\mu_{0}\right)}^{2}.$$

In the equation, *P*_1_ *= N*_1_*/N*_0_ and *P*_2_ = *N*_2_*/N*_0_. *N*_0_ represents the total number of image pixels. *N*_1_ and *N*_2_ represent the number of pixels in the two types of images, respectively. *μ*_0_ represents the mean value of the gray level of the entire image. *μ*_1_ and *μ*_2_ represent the mean value of the gray levels of the two types of images. When the interclass variance *IV* takes the maximum value, the optimal threshold *H* is obtained. We take this threshold as the high threshold of the Canny operator, and we take half of the high threshold as the low threshold.

**Figure 7:**
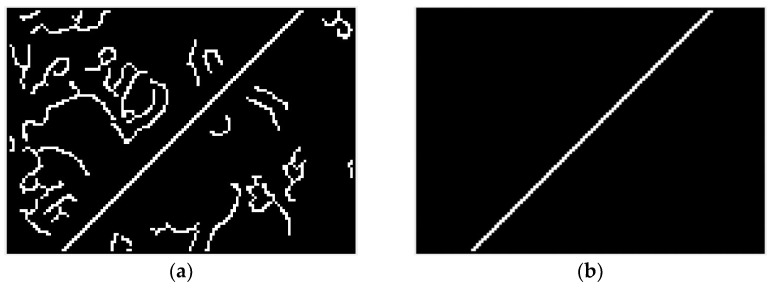
Results of image preprocessing. (**a**) Processing results of the original Canny operator. (**b**) Processing results of the improved Canny operator.

The results derived by the original Canny operator, and the Canny operator based on adaptive filtering and the Otsu method, when processing the noisy images shown in Figure 5 are shown in Figure 7. From Figure 7, we can see that the edge extraction effect of the original Canny operator is poor. The improved Canny operator in this paper has a very good processing effect, and it can remove false edges while retaining slanted-edge information.

In order to verify the accuracy and stability of the improved algorithm in the detection of the slanted edge, this paper simulates and analyzes the detection accuracy of the slanted edge under different SNRs via four methods: the ISO 12233 method, the method combining adaptive filtering and the ISO 12233 method, the Fermi function method (which is currently being used more often), and the method combining the improved Canny operator and the Hough transform. The parameters of the simulated image are the same as those in Figure 5. In order to reduce the random error caused by the added noise of the computer, an image with the same SNR will be detected 1000 times by each method. The relative errors of each method are shown in Table 1.

From the results in Table 1, we can see that the accuracy of detecting the slanted edge using each of the four methods is very high when no noise is added. When noise with different SNRs is added, the accuracy of all four methods is reduced. Among them, the accuracy of the ISO 12233 method is the most affected by noise. After adaptive Gaussian filtering, the ISO 12233 method is less affected by noise. The accuracy of the Fermi function method is higher than that of the ISO 12233 method after adaptive filtering. The improved method in this paper yields the highest accuracy and is the least affected by noise. Therefore, the improved slanted-edge detection algorithm can provide a solid guarantee of the high precision required by Bayer filter color cameras.

### 3.2. Acquisition and Processing of ESF

**Figure 8:**
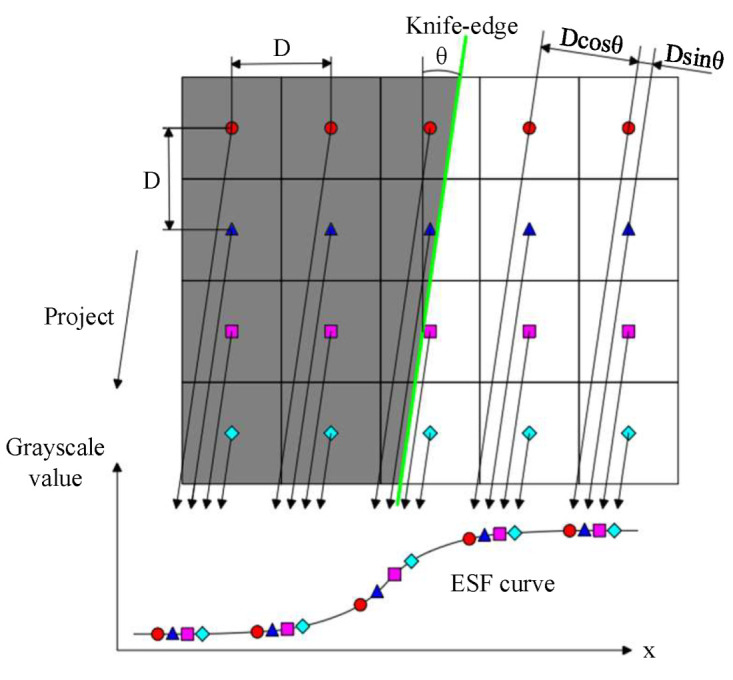
Principle of ESF obtained by projection method.

The ESF can be obtained by processing the image according to the results obtained from the detection of the slanted edge, which is based on the light intensity distribution of the image with the slanted edge. The approach to the acquisition of the ESF can be divided into parametric and non-parametric methods, according to whether a mathematical model is used. The parametric method involves using the function model to directly fit the image data, and this places higher requirements on the model. However, it is not easy to obtain a high-precision function model in practice, and due to the influence of noise factors, the parametric method often has a poor fitting effect or may even encounter non-convergence in the application process. The non-parametric method directly processes the data of each line of the image and has greater adaptability. In this paper, we chose the projection method for ESF acquisition, which is also used in the ISO 12233 method. As shown in Figure 8, the basic principle of the projection method is to use the characteristic of a small phase shift between different scan lines formed by the angle between the slanted edge and the scan direction and project the data in each line in the direction of the slanted-edge; the ESF is then obtained by averaging the data within the same phase shift period.

The following relationship can be obtained from Figure 8:

(6) $$\left\{\begin{array}{ll} m=\frac{1}{\tan\theta } & 0<\theta <\frac{\pi }{4}\\ m=\tan\theta & \frac{\pi }{4}<\theta <\frac{\pi }{2} \end{array}\right.,$$

where *m* represents the sampling rate of the ESF and *θ* represents the angle of the slanted edge. From the above equation, we can see that when the sampling rate of ESF is not an integer, the data in the ESF are uneven, which will affect the accuracy of LSF extraction and *MTF* calculation. In the ISO 12233 method, it is suggested that the sampling rate of the ESF should be an integer, but due to the accuracy of the algorithm and hardware, it is difficult to achieve uniform sampling of the ESF. In addition, the noise will prevent the ESF curve from being smooth, which will reduce the calculation accuracy of the *MTF*. Currently, methods used for smoothing, such as mean filter, median filter, and Gaussian filter, cannot preserve the motion trend of the signal well. Therefore, in order to solve the above problems and realize the uniformization and smoothing of the ESF data, this paper proposes a method combining cubic spline interpolation and SG filtering to process the ESF.

The cubic spline Interpolation algorithm divides two adjacent data points into *n* intervals, and the interpolation function on each interval is a cubic equation. After the ESF curve with continuous curvature is obtained, evenly distributed ESF data can be obtained by uniform sampling on the smooth curve. The calculation method is as follows:

(7) $$S\left(x\right)=\left\{\begin{array}{cc} S_{0}\left(x\right) & x\in \left[t_{0},t_{1}\right]\\ S_{1}\left(x\right) & x\in \left[t_{1},t_{2}\right]\\ \ldots & \ldots \\ S_{n-1}\left(x\right) & x\in \left[t_{n-1},t_{n}\right] \end{array}\right..$$

In the above equation, *S_i_*(*x*) = *a_i_x*^3^ *+ b_i_x*^2^ *+ c_i_x + d_i_*. The undetermined coefficients *a_i_*, *b_i_*, *c_i_*, and *d_i_* on each small interval can be determined according to the continuity condition and the smooth curve condition.

The SG filter divides the ESF data into countless local small areas and performs polynomial least squares fitting on each local area to ensure that the shape of the data curve remains unchanged while filtering out noise. Assuming that the data of a certain local area in the ESF *x*(*n*) are *x*(*i*), *i* = −*m*, …, 0, …, *m*, the *h*-order polynomial is used to fit these local data.

(8) $$f_{i}=e_{0}+e_{1}+e_{2}+\ldots +e_{k}i^{h}=\sum_{k=0}^{h}e_{k}i^{k},h\leq 2m.$$

Using the least squares method to solve the coefficients of the above polynomial, the sum of the squares of the deviations between the fitted curve and the data points is

(9) $$E_{r}={\sum_{i=-m}^{m}\left[f_{i}-x\left(i\right)\right]}^{2}={\sum_{i=-m}^{m}\left[\sum_{k=0}^{h}e_{k}i^{k}-x\left(i\right)\right]}^{2}.$$

When the derivative of *E_r_* is 0, *E_r_* takes the minimum value, and the expression of the function of *f_i_* can be obtained by substitution. The value of the fitted polynomial in the center point coordinates is the resulting value of the SG filter at that center point, and the smoothed result of all ESF data can be obtained by continuously moving the frame of the SG filter. In order to retain the trend of the original ESF curve as much as possible, this paper will use a fifth-order polynomial for fitting, with a frame length of 11.

**Figure 9:**
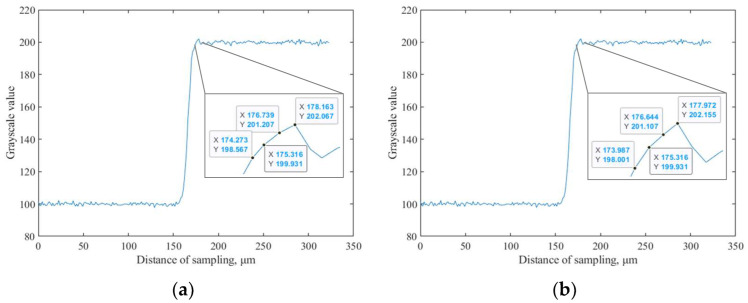

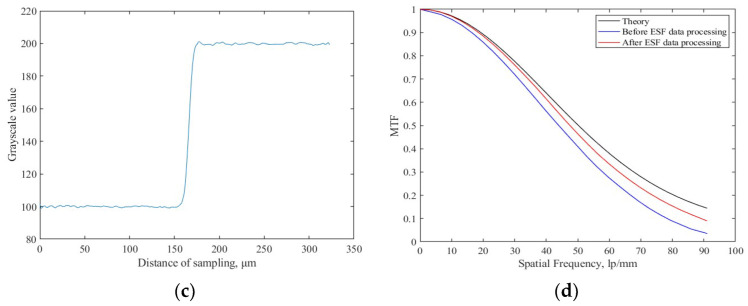
Simulation results when SNR is 20 dB. (**a**) Original ESF data; (**b**) ESF data after cubic spline interpolation; (**c**) ESF data after SG filtering; (**d**) *MTF* calculation results.

In order to verify the effectiveness of the improved ESF processing method using cubic spline interpolation and SG filtering, a simulation analysis is conducted in this paper, and the parameters of the simulated images are shown in Table 2. Firstly, we use the improved method described in the previous section to detect the slanted edge, and then use the ISO 12233 method and the improved ESF processing method in this paper to process the ESF data; finally, the accuracy of the different methods can be evaluated by the root mean squared error (RMSE) of the calculated and theoretical values of the *MTF*. The simulation results yielded when the SNR is 20 dB are shown in Figure 9, and the RMSEs of *MTF*s of different methods with different SNRs are shown in Table 3.

It can be seen from Figure 9 that the original distribution of ESF data is non-uniform, and the data points can be uniformly distributed using cubic spline interpolation. Using SG filtering can smooth the ESF curve under the premise of ensuring the trend of the curve. As shown in Table 3, when the SNR is 15 dB, the ISO 12233 method fails to complete the calculation of the *MTF* curve due to the serious influence of noise, even though the detection algorithm with the improved slanted edge described in the previous section is used. After ESF is processed by the combination of cubic spline interpolation and SG filtering, as proposed in this paper, the calculation of the *MTF* curve is successfully completed when the SNR is 15 dB. Moreover, the method proposed in this paper achieves greater accuracy when the SNR is different. Therefore, the processing method of ESF proposed in this paper not only broadens the scope of application of the slanted-edge method but also improves the measurement accuracy of *MTF*.

### 3.3. Acquisition and Processing of LSF

Discrete data with uniform distribution will be obtained after processing the ESF in the previous section, and then the LSF can be obtained from the difference operation. Although the SG filtering of the ESF in the previous section reduces the noise to a certain extent, the differential operation will expand the influence of the noise, so the influence of the residual noise on the *MTF* results cannot be ignored. Therefore, the LSF needs to be smoothed or fitted to reduce the effect of noise. The LSF is smoothed using the Hamming window in the ISO 12233 method; the expression of the Hamming window is

(10) $$hw\left(n\right)=\left\{\begin{array}{cc} 0.54-0.46\cos\frac{2\pi n}{N} & 0\leq n\leq N\\ 0 & else \end{array},\right.$$

where *N +* 1 represents the length of the window. The Hamming window can effectively reduce the oscillation noise caused by the differential operation, but when the noise is large, the improvement in accuracy yielded using the Hamming window for smoothing is limited. Since PSF is the intensity distribution function of single-point imaging, LSF involves the sampling and superposition of PSF in a certain direction, and the distribution of PSF can be expressed by the Gaussian function; this paper proposes a Gaussian function fitting to process LSF.

(11) $$y=A\cdot \exp\frac{-{\left(x-\mu \right)}^{2}}{2\sigma^{2}}.$$

Here, *A* represents the peak value, *μ* represents the mean value, and *σ* represents the variance.

**Figure 10:**
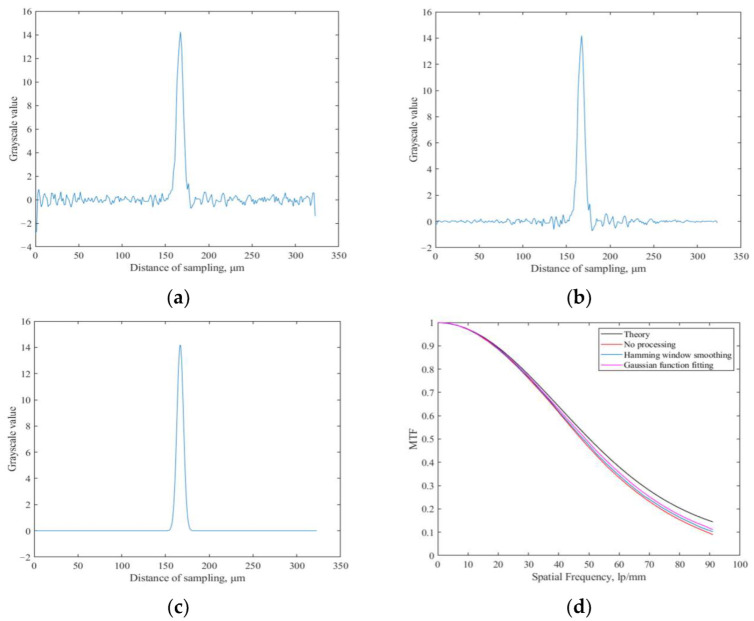
Simulation results of different processing methods of LSF. (**a**) LSF before processing; (**b**) LSF after Hamming window smoothing; (**c**) LSF after Gaussian function fitting; (**d**) *MTF* calculation results.

Hamming window smoothing and Gaussian function fitting are separately performed on the results shown in Figure 9c, and then the *MTF* is obtained by Fourier transform. The simulation results are shown in Figure 10.

It can be seen from Figure 10a that the smoothing of the Hamming window can reduce the influence of noise to a certain extent, but when the noise is large, the processing effect of the Hamming window is limited. Using the Gaussian function fitting method proposed in this paper to process LSF, the obtained accuracy of the *MTF* is relatively high.

### 3.4. Process of the Improved Algorithm

After the improvement of the slanted-edge method shown in the previous three sections, the process of the improved algorithm can be divided into six steps:

1. After the image is taken by the Bayer filter color camera, the image with the slanted edge in the appropriate position is selected;
2. The method combining the improved Canny operator and the Hough transform is used to complete the detection of the slanted edge and to fit the equation of the slanted edge;
3. Based on the information of the detected slanted edge, ESF data are obtained by using the projection method;
4. The method of combining cubic spline interpolation and SG filtering is used to complete the uniformization and smoothing of ESF data;
5. The difference operation is performed on the ESF to obtain the LSF, and the Gaussian function is fitted to the LSF to derive the smoothed LSF;
6. LSF is Fourier transformed to derive the *MTF*.

### 3.5. Accuracy Analysis of the Improved Algorithm

**Figure 11:**
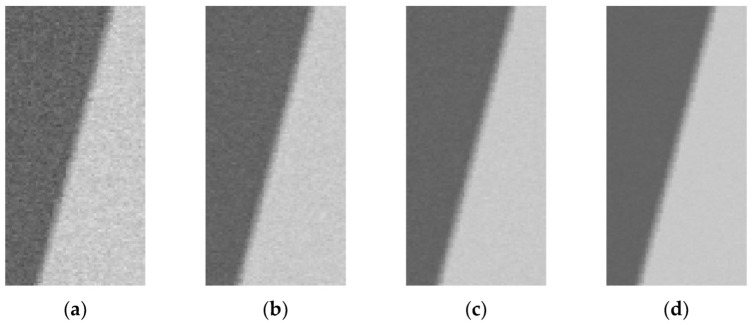
Simulated images with different SNRs. (**a**) SNR equal to 15 dB; (**b**) SNR equal to 20 dB; (**c**) SNR equal to 25 dB; (**d**) SNR equal to 30 dB.

**Figure 12:**
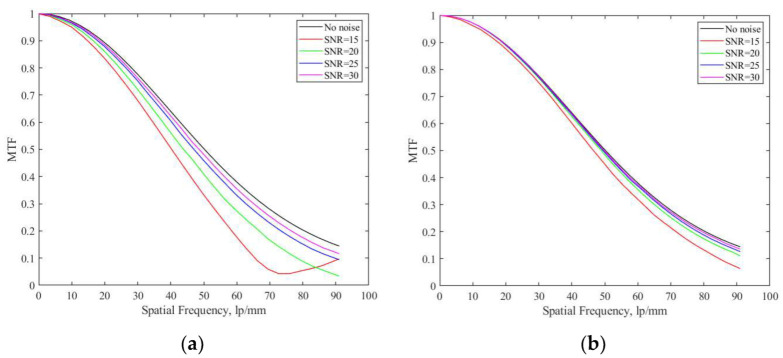
*MTF* calculation results of different methods at different SNRs. (**a**) Results of the ISO 12233 method; (**b**) results of the improved slanted-edge method.

In order to verify the overall accuracy and stability of the improved slanted-edge method, this paper uses the ISO 12233 method and the improved slanted-edge method to simulate and analyze images with different SNRs. The parameters of the simulated image are the same as those in Table 2, and the simulated images with different SNRs are shown in Figure 11. The calculation result of *MTF* is shown in Figure 12, and the mean square error between the *MTF* values obtained by the two methods and the theoretical *MTF* value is shown in Table 4.

It can be seen from Figure 12 that when the SNR of the image is 15 dB, the *MTF* curve obtained by the ISO 12233 method is aliased before the Nyquist frequency, which is caused by the presence of large noise and the insufficiency of the algorithm’s precision. The improved slanted-edge method shown in this paper can successfully complete the measurement of *MTF* when the SNR is 15 dB. It can be seen from Figure 12 and Table 4 that the accuracy of the improved slanted-edge method in this paper is higher than that of the ISO 12233 method. Therefore, the improved method has higher precision and wider adaptability and provides an important guarantee in the measurement of the *MTF* of a Bayer filter color camera.

## 4. Experiments

### 4.1. Calibration of Weighting Factors of RGB Three Primary Colors

In order to realize the accurate measurement of the *MTF* of the full frequency band of the color camera, this paper first uses the bilinear interpolation algorithm to perform color synthesis on the original image obtained by the Bayer filter detector and obtains the image information of the red, green, and blue channels. Then, the *MTF* values of the three channels are determined separately by adopting the method of channel division. Finally, using the weighting factors of the three primary colors measured in the test, the *MTF* of the color camera is obtained by weighting.

The spectral range of the color camera subjected to *MTF* measurement in this paper is the visible light band, so it is necessary to test and measure the weighting factors of the three primary colors of the camera in this band. The weighting factors of the three primary colors are related to the spectral distribution of the light source and the response of the detector. Assuming that the spectral distribution of the light source is *L(λ)*, and the response of the three primary colors of the detector is *Q(λ)*, the calculation of the weighting factors of the three primary colors can be expressed as

(12) $$w=\int_{380}^{780}L\left(\lambda \right)Q\left(\lambda \right)d\lambda .$$

**Figure 13:**
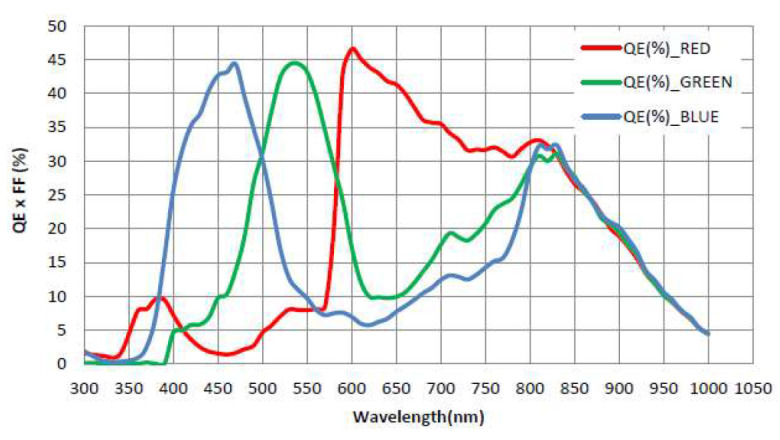
Quantum efficiency (QE) curve of the detector.

The QEs of the three primary colors of the Bayer filter detector used in the test camera in this paper are shown in Figure 13; the data come from the official website of Gpixel. QE is related to factors such as wavelength, material, and packaging process. One must comprehensively consider multiple parameters in the selection of detectors, such as QE, pixel size, and number of pixels. Therefore, the final choice of the detector will be relatively reasonable.

**Figure 14:**
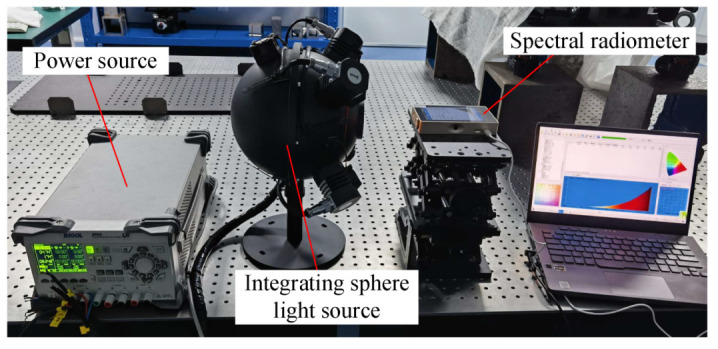
Test site for spectral radiation of light source.

**Figure 15:**
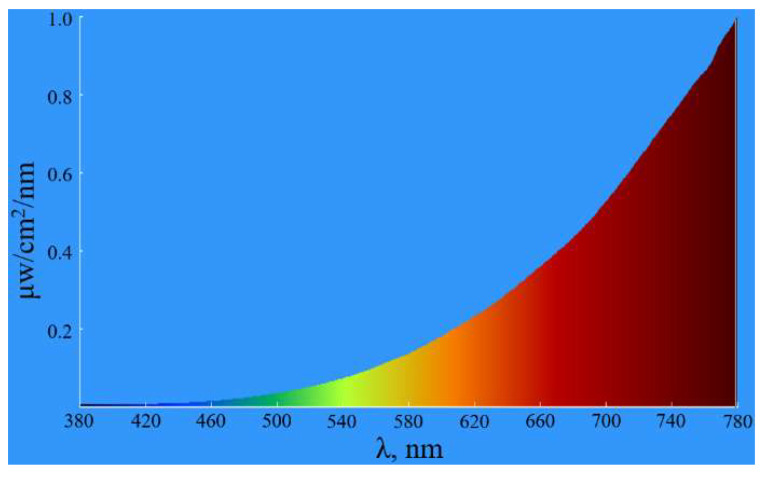
Distribution curve of the spectral radiant energy of the light source.

The light source used in this experiment is a halogen lamp. Since the spectral range of the detected camera is 380–780 nm, it is necessary to use a spectroradiometer to measure the spectral distribution of the light source at 380–780 nm. The test site is shown in Figure 14, and the test results are shown in Figure 15.

According to the data in Figure 14 and Figure 15, and using Equation (12), the final weighting factors obtained after the normalization calculation are 0.503, 0.293, and 0.204 for the three primary colors, respectively. The weighting calculation of the *MTF* of a color image can be expressed as:

(13) $$\begin{array}{ll} MTF & =w_{R}\cdot MTF_{R}+w_{G}\cdot MTF_{G}+w_{B}\cdot MTF_{B}\\ & =0.503\cdot MTF_{R}+0.293\cdot MTF_{G}+0.204\cdot MTF_{B} \end{array},$$

where *w_R_*, *w_G_*, and *w_B_* are the weighting factors for red, green, and blue, respectively.

### 4.2. Measurement of MTF

In order to verify the accuracy of the improved algorithm in this paper, the *MTF* of a Bayer filter color space camera is measured. The measurement device includes an integrating sphere light source, a knife-edge target, a collimator, and a Bayer filter color space camera. The parameters of the measuring device are shown in Table 5.

**Figure 16:**
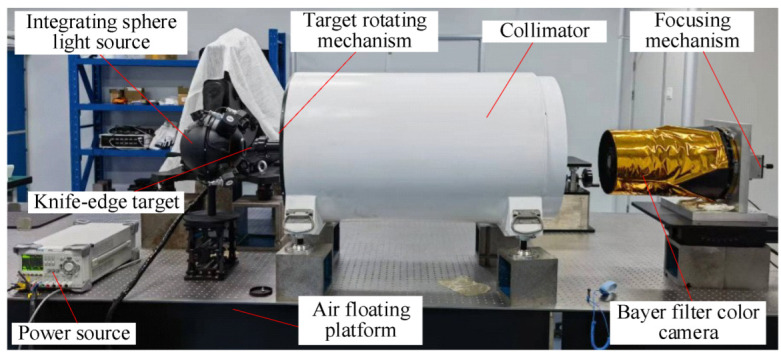
Test devices for the *MTF* test of a color space camera.

**Figure 17:**
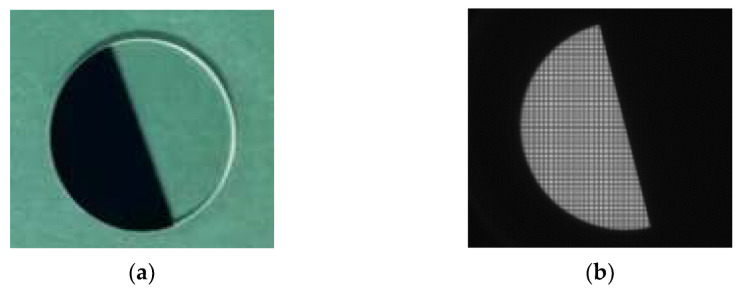
Knife-edge target. (**a**) Physical image; (**b**) acquired image.

As shown in Figure 16, the positions of the integrating sphere, the collimator, and the camera are adjusted on the air-floating optical platform such that the optical path is coaxial. Multiple dark background images are acquired continuously, and the average value is taken as the environmental background noise value. Using the focusing mechanism to move the detector to the designated theoretical position, the image is acquired when the view is clearest. The target rotation mechanism can be used to obtain images with different angles of slanted edges. The knife target and the acquired original image are shown in Figure 17.

**Figure 18:**
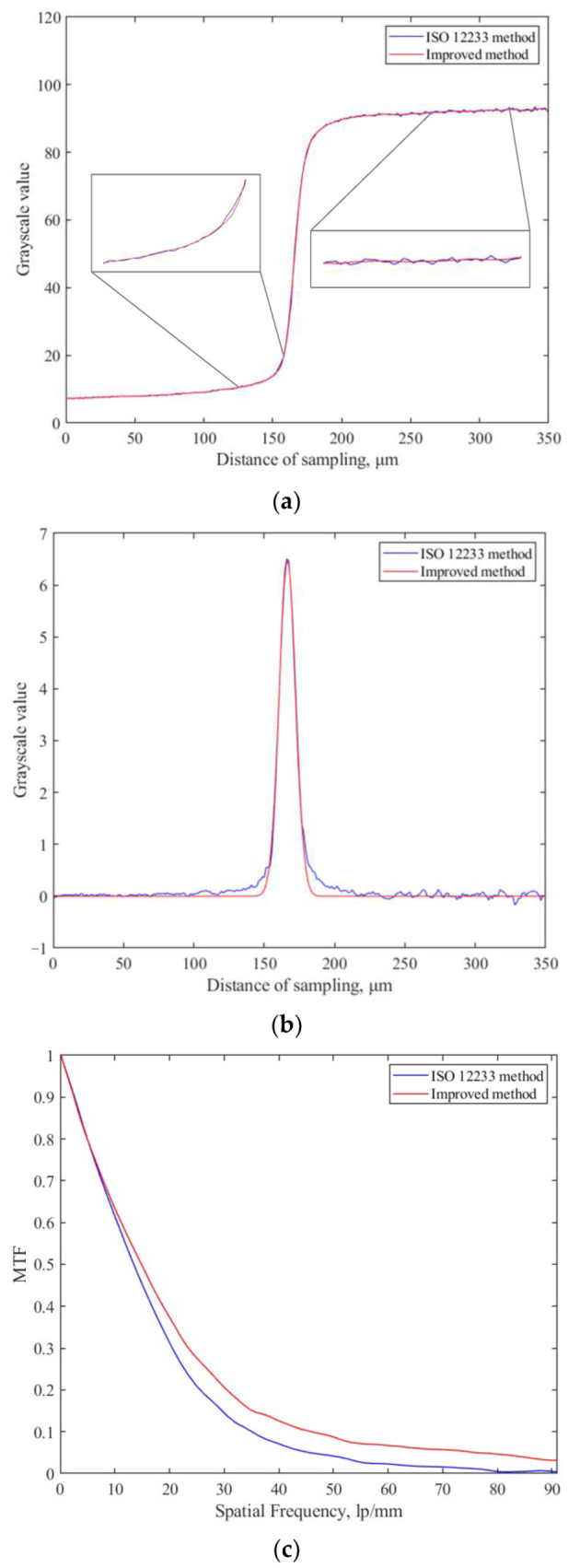
Test results. (**a**) Results of ESF; (**b**) results of LSF; (**c**) results of *MTF*.

The RGB image of the entire camera surface can be obtained using the bilinear interpolation algorithm, and the *MTF* of the color image can be obtained by weighting with the three primary color weight factors obtained in the previous section. The ISO 12233 method and the improved slanted-edge method are used to measure the *MTF* values of images with two different slanted-edge angles. The measurement results are shown in Figure 18 and Table 6.

*f_N_* represents the Nyquist frequency. Figure 18 and Table 6 show that the ESF and LSF obtained by the improved slanted-edge method are smoother and less affected by noise. The *MTF* curve obtained by the ISO 12233 method is aliased when it is less than the Nyquist frequency, and the improved method successfully completes the *MTF* measurement. Therefore, the improved slanted-edge method shown in this paper has higher precision and wider applicability, and it is more suitable for the measurement of the *MTF* of Bayer filter color cameras.

## 5. Conclusions

In order to solve the difficult problem of the *MTF* detection of Bayer filter color cameras, this paper realizes the high-precision measurement of full-band *MTF* by improving the traditional slanted-edge method. First, this paper analyzes the causes of image quality degradation encountered in Bayer filter color cameras. Then, the improvement of the slanted-edge method is carried out via three steps: the detection of the slanted edge, the acquisition and processing of the ESF, and the acquisition and processing of LSF. The Canny operator is improved to achieve the optimal selection of scale parameters by constructing an adaptive filtering function, and the optimal selection of thresholds in the Canny operator is achieved using the Otsu method. A combination of the improved Canny operator and the Hough transform is proposed for the detection of the slanted edge, which improves the resistance of the algorithm to noise. A combined method of cubic spline interpolation and SG filtering is proposed to process the ESF data, which reduces the influence of the non-uniform distribution of ESF data and noise on the accuracy. Gaussian function fitting is proposed to process the LSF, which further reduces the influence of noise on the *MTF* calculation results. Finally, the accuracy of the improved slanted-edge method is verified by testing the *MTF* measurement of a certain type of Bayer filter color space camera. The simulation and test results show that the improved slanted-edge method in this paper has the characteristics of high accuracy, strong stability, and wide applicability and can thus effectively solve the problems encountered by Bayer filter color cameras in *MTF* measurement.

## Figures and Tables

**Table 1:** Relative errors in the detection of the slanted edge by different methods.

	Methods	ISO 12233 Method	Adaptive Filtering + ISO 12233	Fermi Function Method	Improved Method
SNR/dB					
15		0.0384	0.0144	0.0132	0.0061
20		0.0228	0.0085	0.0063	0.0010
25		0.0022	0.0013	0.0009	0.0006
30		0.0008	0.0005	0.0004	0.0003

**Table 2:** Parameters of the simulation image.

Parameters	Values
Size of image	120 × 60 pixels
Size of pixel	5.5 μm
Angle of slanted-edge	15°
DN * value of the bright side	200
DN value of the dark side	100
Variance of the Gaussian function as a low-pass filter	2

* DN represents the digital number value.

**Table 3:** RMSE of *MTF* for different processing methods of ESF.

	Methods	ISO 12233 Method	After ESF Data Processing
SNR/dB			
15		—	0.010296
20		0.006592	0.001212
25		0.001387	0.000182
30		0.000376	0.000085

**Table 4:** RMSE of *MTF* calculation via different methods.

	Methods	ISO 12233 Method	Improved Method
SNR/dB			
15		—	0.002452
20		0.007035	0.000675
25		0.001465	0.000101
30		0.000399	0.000027

**Table 5:** Parameters of test devices.

Test Devices	Parameters	Type/Model
Integrating sphere	Spherical diameter: 200 mm	LY-FP-JFQ200
	Diameter of light outlet: 50 mm	
Slanted-edge target	Diameter: 25 mm	Self-developed
	Thickness: 3 mm	
Collimator	Focal length: 15,000 mm	Self-developed
	Aperture: 400 mm	
Bayer filter detector	Spectral range: 380~780 nm	GMAX1205
	Pixel size: 5.5 μm × 5.5 μm	
Optical lens	Focal length: 1850 mm	Self-developed
	Aperture: 210 mm	

**Table 6:** Comparison of *MTF* results of different methods.

Methods	Detection Result of *θ*	*MTF* of *f_N_*/2	*MTF* of *f_N_*
ISO 12233 method	19.7961	0.0501	—
Improved method	19.9594	0.1017	0.0310

## Data Availability

Not applicable.

## References

[1] Bayer B.E. (1976). Color Imaging Array. U.S. Patent.

[2] Bae T.W. (2020). Image-quality metric system for color filter array evaluation. PLoS ONE.

[3] Feltz J.C., Karim M.A. (1990). Modulation transfer function of charge-coupled devices. Appl. Opt..

[4] Kabir S., Leigh L., Helder D. (2020). Vicarious Methodologies to Assess and Improve the Quality of the Optical Remote Sensing Images: A Critical Review. Remote Sens..

[5] Xu L., Yan C., Gu Z., Li M., Li C. (2019). Analysis of Dynamic Modulation Transfer Function for Complex Image Motion. Appl. Sci..

[6] Metz C.E., Strubler K.A., Rossmann K. (1972). Choice of line spread function sampling distance for computing the *MTF* of radiographic screen-film systems. Phys. Med. Biol..

[7] Morishita J., Doi K., Bollen R., Bunch P.C., Hoeschen D., Sirand-rey G., Sukenobu Y. (1998). Comparison of two methods for accurate measurement of modulation transfer functions of screen-film systems. Med. Phys..

[8] Sitter D.N., Goddard J.S., Ferrell R.K. (1995). Method for the measurement of the modulation transfer function of sampled imaging systems from bar-target patterns. Appl. Opt..

[9] Yamazaki T., Nokita M., Hayashida S., Inoue H. (2004). A method to measure the presampling *MTF* using a novel edge test device and algorithm. Proc. SPIE Int. Soc. Opt. Eng..

[10] Duan Y.-X., Liu S.-K., Chen Y.-Q., Xue X., Zhao J.-K., Gao L.-M. (2017). A method to measure the modulation transfer function of Bayer filter color camera. Acta Phys. Sin..

[11] Zhang S., Wang F., Wu X., Gao K. (2023). *MTF* Measurement by Slanted-Edge Method Based on Improved Zernike Moments. Sensors.

[12] (2000). Photography-Electronic Still-Picture Cameras-Resolution Measurements.

[13] Canny J. (1986). A Computational Approach to Edge Detection. IEEE Trans. Pattern Anal. Mach. Intell..

[14] Mcilhagga W. (2011). The Canny Edge Detector Revisited. Int. J. Comput. Vis..

[15] Otsu N. (1979). A Threshold Selection Method from Gray-Level Histograms. IEEE Trans. Syst. Man Cybern..

[16] Hough P.V.C. (1962). General purpose visual input for a computer*. Ann. N. Y. Acad. Sci..

[17] Duda R.O., Hart P.E. (1972). Use of the Hough transformation to detect lines and curves in pictures. Commun. ACM.

[18] Dongfeng R., Qiubing W., Fujun S. (2016). A fast and effective algorithm based on improved hough transform. J. Indian Soc. Remote Sens..

[19] Righini A. (1972). Modulation Transfer Function for Solar Telescopes and Atmosphere Turbulence. Sol. Phys..

[20] Yotam E., Ephi P., Ami Y. (2007). *MTF* for Bayer Pattern Color Detector. Signal Processing, Sensor Fusion, and Target Recognition XVI.

[21] Volyar A.V., Egorov Y.A., Rubass A.F., Fadeeva T.A. (2004). Fine Structure of “White” Optical Vortices in Crystals. Tech. Phys. Lett..

[22] Wang C., Liu C., Zhang Y., Hu H., Liu S. (2022). Image blur analysis and elimination algorithm for an *MTF* test target of a space camera. Appl. Opt..

[23] Li L., Li Z., Wang Z., Jiang Y., Shen X., Wu J. (2023). On-Orbit Relative Radiometric Calibration of the Bayer Pattern Push-Broom Sensor for Zhuhai-1 Video Satellites. Remote Sens..

[24] Xia M., Wang C., Ge W. (2019). Weights-Based Image Demosaicking Using Posteriori Gradients and the Correlation of R–B Channels in High Frequency. Symmetry.

[25] Cok D.R. (1987). Signal Processing Method and Apparatus for Producing Interpolated Chrominance Values in a Sampled Color Image Signal. U.S. Patent.

[26] Hibbard R.H. (1995). Apparatus and Method for Adaptively Interpolating a Full Color Image Utilizing Luminance Gradients. U.S. Patent.

